# Innovative geopolymer-based cold asphalt emulsion mixture as eco-friendly material

**DOI:** 10.1038/s41598-023-44630-5

**Published:** 2023-10-13

**Authors:** Anmar Dulaimi, Shakir Al Busaltan, Md Azree Othuman Mydin, Dong Lu, Yasin Onuralp Özkılıç, Ramadhansyah Putra Jaya, Arman Ameen

**Affiliations:** 1 College of Engineering, University of Warith Al-Anbiyaa, Karbala, 56001 Iraq; 2 School of Civil Engineering and Built Environment, Liverpool John Moores University, Liverpool, L3 2ET UK; 3 Department of Civil Engineering, College of Engineering, University of Kerbala, Karbala, 56001 Iraq; 4 School of Housing, Building and Planning, Universiti Sains Malaysia, 11800 Penang, Malaysia; 5 School of Civil Engineering, Harbin Institute of Technology, Harbin, 150090 People’s Republic of China; 6 Key Lab of Structures Dynamic Behavior and Control of the Ministry of Education, Harbin Institute of Technology, Harbin, 150090 People’s Republic of China; 7 Department of Civil Engineering, Faculty of Engineering, Necmettin Erbakan University, 42000 Konya, Turkey; 8 Department of Civil Engineering, Lebanese American University, Byblos, 1102-2801 Lebanon; 9 Faculty of Civil Engineering Technology, Universiti Malaysia Pahang Al-Sultan Abdullah, 26300 Kuantan, Malaysia; 10 Department of Building Engineering, Energy Systems and Sustainability Science, University of Gävle, 801 76 Gävle, Sweden

**Keywords:** Polymers, Environmental impact

## Abstract

In recent years, there has been a growing interest in cold asphalt emulsion mixture (CAEM) due to its numerous advantages, including reduced CO_2_ emissions, energy savings, and improved safety during construction and application. However, CAEM has often been considered inferior to hot mix asphalt (HMA) in terms of performance. To address this issue and achieve desirable performance characteristics, researchers have been exploring the modification of CAEM using high-cost additives like ordinary Portland cement. In this study, the focus was on investigating the effects of utilizing waste alkaline Ca(OH)_2_ solution, ground granulated blast-furnace slag (GGBFS), and calcium carbide residue (CCR) as modifiers to enhance the properties of CAEM. The aim was to develop an innovative geopolymer geopolymer-based cold asphalt emulsion mixture (GCAE). The results of the study revealed that the use of waste alkaline Ca(OH)_2_ solution led to an increase in early hydration, which was confirmed through scanning electron microscopy. Furthermore, the experimental findings demonstrated that waste alkaline Ca(OH)_2_ solution significantly contributed to the rapid development of early-age strength in GCAE. As a result, GCAE showed great potential for utilization in pavement applications, particularly for roads subjected to harsh service conditions involving moisture and temperature. By exploring these alternative modifiers, the study highlights a promising avenue for enhancing the performance of CAEM and potentially reducing the reliance on expensive additives like ordinary Portland cement. The development of GCAE has the potential to offer improved performance and durability in pavement applications, thus contributing to sustainable and efficient road infrastructure.

## Introduction

Global warming stands as one of the most critical challenges we face today, and the construction industry plays a significant role in contributing to greenhouse gas emissions. In response to this issue, various sectors are adopting sustainable practices and products, and the highway industry is actively taking steps to minimize its carbon footprint. While hot mix asphalt (HMA) remains widely utilized for pavement construction due to its lower initial cost and superior performance^1,2^, it is produced at high temperatures (> 140 °C), leading to adverse environmental impacts such as the release of greenhouse gases and hazardous fumes^3–5^.

Cold asphalt emulsion mixture (CAEM) refers to a type of cold mix asphalt (CMA) that is produced by combining emulsified bitumen with unheated aggregates. This method eliminates the need for aggregate heating, making CAEM cost-effective and environmentally friendly^6,7^. Although CMA offers advantages in terms of cost and environmental impact compared to warm mix asphalt (WMA) and hot mix asphalt (HMA), it falls short in terms of strength and stability. The Marshall Stability values of unmodified CMA are significantly lower than those of HMA and WMA^8,9^. Additionally, CMA tends to have higher air voids compared to HMA, which can impact its service life negatively^10^. As a result, CMA is typically used for footpath reinstatement and low-to-moderate-traffic roadways^11–15^.

Numerous studies have been carried out to see how different types of filler materials affect CMA performance. Filler materials, typically those that pass through a sieve size of 75 μm, are commonly used in asphalt mixes. Furthermore, Ordinary Portland Cement (OPC) has been extensively employed as a hydraulic binder to produce CAEMs that exhibit satisfactory performance^16,17^. The cement-modified CMA has increased strength and resistance to water damage, temperature sensitivity, permanent deformation and creep^18,19^. Wang et al.^20^ demonstrated that the cement paste has alkaline nature, so the breakdown of the cationic bitumen emulsion can be accelerated by raising the pH due to the presence of OPC in the CMA. This allows the flocculation of the asphalt emulsion and makes a rapid coalescence due to the increase in the dissociation rate of the emulsifier on the asphalt droplets. However, the manufacture of one ton of OPC emits about one ton of carbon dioxide and consumes roughly 2.5 tonnes of materials^21^, accounting for about 7% of total greenhouse gas emissions worldwide^22^. As a result, reports of the manufacture of CAEMs using industrial by-products or solid wastes began to circulate^23,24^.

Industrial by-products and solid waste are becoming a growing concern worldwide, leading to significant environmental and economic challenges. To address this issue, there is an increasing trend in developing cold asphalt emulsion mixture (CAEM) using different by-products and waste materials. One such by-product is ground granulated blast-furnace slag (GGBFS), which is obtained through the grinding of blast-furnace slag, a residue of the iron-making industry. For many years, GGBFS has been widely utilized in Portland cement concrete as a secondary cementitious material (SCM), either as a mineral additive or as a component of blended cement, often replacing 35–65% of ordinary Portland cement (OPC)^25^. It was demonstrated by Ellis et al.^26^ that in comparison to reference mixes, adding GGBFS to CAEM mixes increased long-term mechanical performance. In addition, comparison research was carried out with two types of fillers, namely cement and GGBFS (each of 2% by the total mass of the mix), and performance was measured in terms of Marshall characteristics^27^. However, the GGBFS had a greater Marshall Stability, the cement had a higher density and fewer air voids. The inclusion of a low cement percentage (1%) in combination with ground granulated blast-furnace slag (GGBFS) and fly ash has been shown to enhance the mechanical properties and durability of cold mix asphalt (CMA), resulting in performance comparable to high cement content HMA and CMA^28^. Bijen^29^ also found that GGBFS, with its high lime content, acts as a latent hydraulic cement that requires activation. Since GGBFS exhibits a slower hydration rate compared to Portland cement, activators for example alkalis, Portland cement, or lime are used to accelerate the process^30^.

Calcium carbide residue (CCR) is a waste product generated during the hydrolysis of calcium carbide to produce acetylene gas. Due to its high alkalinity and significant metal content, CCR seriously harms the environment^31^. The high alkalinity of CCR makes it appropriate for activating GGBFS, thereby enhancing its reactivity during the early stages^32,33^.

On the other hand, because geopolymers have environmentally friendly features, they can partially or completely replace OPC, and their solid precursors include fly ash, metakaolin, slag, and other materials^34–36^.

Geopolymers have been attracting the attention of researchers due to their feasibility, low cost, and environmentally friendly nature, making them viable alternatives to organic polymers in road construction^37^. The strength development of recycled asphalt pavement using fly ash geopolymers has been studied as a potential road construction material. Hoy et al.^38^ further confirm the potential of these geopolymers as alternative stabilized pavement materials in their research work. The incorporation of recycled asphaltic concrete aggregate can be advantageous in enhancing the performance of geopolymer concrete. Despite a minor reduction in compressive strength, it offers improved resistance against surface abrasion and sulfuric acid. By enhancing sulfuric acid resistance and surface abrasion while maintaining reasonable compressive strength, the use of recycled asphaltic concrete aggregate becomes beneficial in improving the overall performance of geopolymer concrete^39^. Meng et al.^40^ investigated the microscopic and mechanical properties of geopolymer-modified asphalt binders and asphalt mixtures. The geopolymer was produced by using alkali activators and aluminosilicate components, which were then employed as asphalt modifiers. Their findings demonstrate that, with the exception of ductility, the modified binders' fundamental characteristics and high-temperature performance improved. It was also reported that geopolymer has been shown to be a promising ingredient for creating warm mix asphalt with excellent performance and significant advantages where the optimal dose of geopolymer additive by weight of bitumen was 6%, while the optimum mixing temperature for warm mix asphalt was around 140 °C^41^. It was found that crushed rock-based alkaline-activated materials, with an optimized mixture comprising a 5 M NaOH concentration, an SS/SH ratio of 1.00, and a liquid alkaline-to-binder (L/B) ratio of 0.5, showed potential for use in roadway applications. Paste samples, cured at room temperature, exhibited an early compressive strength of 3.82 MPa at 3 days of age^42^. Previous study showed that non-OPC binder, based on a hybrid material concept, holds significant potential as an environmentally friendly choice for road construction and rehabilitation in the emerging era of our low-carbon society^43^.

The traditional CMA, characterized by modest early-age strength performance and inadequate water stability, poses practical limitations in meeting the requirements of rapid open traffic, particularly from an engineering performance standpoint. The objective of this study is to present a new approach for developing CAEM using the geopolymer technique as a sustainable pavement material. To investigate the strength development of CAEM geopolymer, a series of extensive laboratory experiments were conducted.

The use of CAEM with GGBFS-CCR based geopolymer by incorporating waste Ca(OH)_2_ solution, a sustainable alternative to HMA, in road pavement has a substantial influence on pavement applications. The durability and mechanical performance of CAEM have been greatly enhanced by the application of various additives, such as OPC. However, such additives could come at a higher starting cost and even increase transportation costs. Additionally, GGBFS, CCR and waste Ca(OH)_2_ solution are produced as waste material, creating environmental and health risks. The large quantities of waste produced pose significant challenges in terms of disposal and create engineering hurdles for their utilization in civil construction. As a consequence, there has been a growing interest in incorporating these waste materials in the development of a novel CAEM.

## Materials and method

### Aggregate

**Figure 1:**
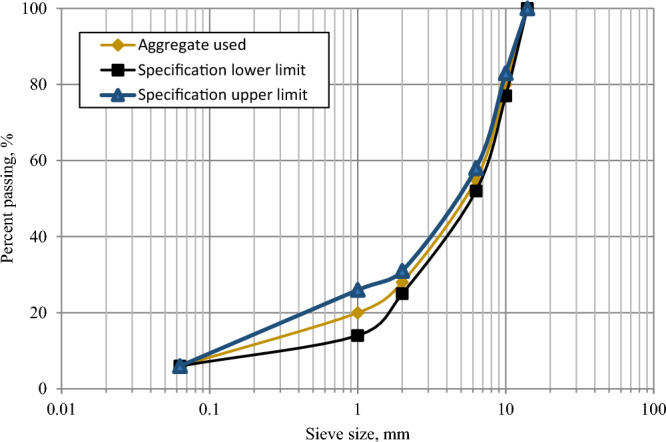
AC 14 mm Gradation.

According to BS EN 933-1^44^, a close-graded, surface coarse gradation of 14 mm was selected, which complied with BS EN standards (Fig. 1). This particular gradation is widely used for producing bituminous mixes^45^. The coarse aggregates used in both the CAEMs and HMA consist of crushed granite with the following properties: water absorption of 0.7%, bulk particle density of 2.60 Mg/m^3^, and apparent particle density of 2.65 Mg/m^3^. On the other hand, the fine aggregate possesses water absorption of 1.5%, a bulk particle density of 2.53 Mg/m^3^, and an apparent particle density of 2.64 Mg/m^3^.

### Bitumen emulsion and asphalt

At room temperature, the emulsion has a decreased viscosity, making it a good CAEMs binding material. For all of the CAEMs, a cationic, slow-setting bitumen emulsion (C50B4) was chosen as the binder. This bitumen emulsion has a penetration grade of 40/60 and is made up of 50% base bitumen and supplied by Jobling Purser of Newcastle, UK.

### asphalt

Two HMAs were included in this study for comparison purposes. These mixtures have the same aggregate type and grade as the CAEMs. The soft grade penetration (142 pen) has softening point and density (at 25 °C) of 51.5 °C and 1.02 g/cm^3^, while hard grade penetration (49 pen) has softening point and density (at 25 °C) of 43.5 °C and 1.04 g/cm^3^, respectively. 5.1% optimum binder content by aggregate weight was used in this study.

### Alkaline activator

To increase the pH concentration of the hydration mediums and facilitate the breaking and dissolution of glassy phases in the mixture, a waste Ca(OH)_2_ solution with high alkalinity was utilized as an alkaline activator. The pH of this solution, which is discharged from CCR's dewatering process, is 13.8. The 3 percent pre-wetting water content was completely replaced with the waste alkaline Ca(OH)_2_ solution.

The advancement of technology in the development of a high-strength cold bitumen emulsion mixture (CBEM) is attributed to the research conducted by Dulaimi et al.^46^. Their study aimed to create a novel CBEM with rapid curing properties. Their findings revealed that by activating a binary blended filler with a waste alkaline NaOH solution, the water susceptibility, mechanical properties, and thermal sensitivity of the CBEM were significantly enhanced.

### Selected fillers

In this study, ground granulated blast-furnace slag (GGBFS) and calcium carbide residue (CCR) were selected as fillers, while traditional limestone filler (TLF) was used as a conventional mineral filler. The aim was to investigate and assess the full potential of these materials by examining their key physical, chemical, mineralogical, and morphological characteristics. Calcium carbide residue (CCR) is a by-product of acetylene synthesis, consisting mainly of Ca(OH)_2_. It is estimated that approximately 74 g of CCR is produced when 64 g of acetylene is synthesized^47^. CCR is produced as a by-product during the industrial production of acetylene gas. The manufacturing process involves the reaction of calcium carbide (CaC_2_) with water, resulting in the formation of acetylene gas (C_2_H_2_), heat, and lime slurry (Ca(OH)_2_). The finished product is a slurry of sludge. The slurry converts into a solid-state residue when the high alkalinity water is fully released, making landfill disposal an expensive and complex task.

**Figure 2:**
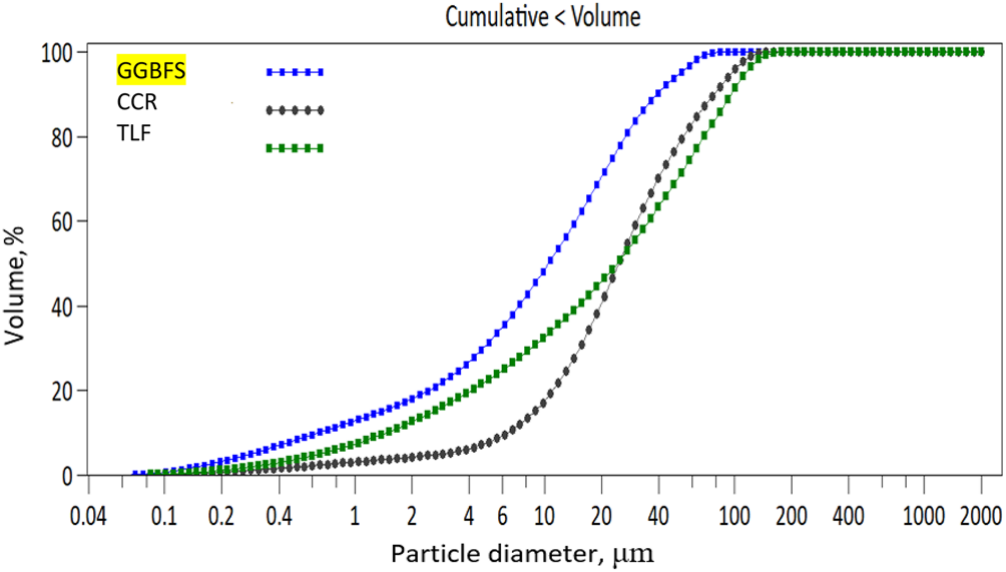
Fillers' particle size distribution.

In 2014, the global production of acetylene gas reached 500,000 tonnes, resulting in the generation of 1,423,000 tonnes of CCR as waste. It is projected that the production of CCR will continue to increase by 3% until 2020^48^. The CCR was in the form of large, moist bricks that required breaking into smaller pieces and drying in an oven at 110 °C for one day. In order to avoid particle agglomeration, which can have a detrimental effect on the quality of the ground CCR, the resulting lumps underwent additional processing in a mechanical grinder. The grinding process involved low-energy, intense, dry agitation for a duration of 15 min. In the GGBFS powder, the particle size distribution shows that over 90% of the particles are less than 40 μm, whereas the CCR’s predominant particle size is practically smaller than 25 μm. When exposed to water, the CCR particles' fineness has a significant impact on how quickly they hydrate. The milling process of the CCR lasted for 15 min, resulting in an average grain size (D50) of 24.6 μm. Figure 2 displays the particle size distribution of the GGBFS, CCR, and TLF, indicating that 90% of the TLF particles were able to pass through an 80 μm sieve. The dominant particle size of the CCR is predominantly below 25 μm, whereas approximately 90% of the GGBFS particles have a size smaller than 40 μm.

**Figure 3:**
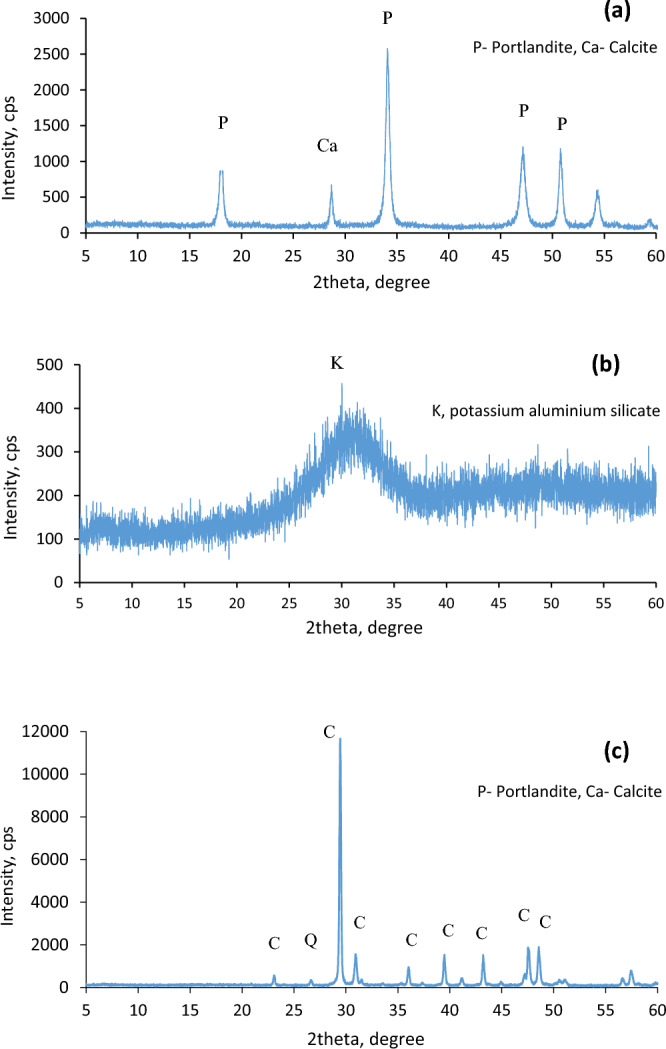
Powder XRD pattern of (**a**) CCR, **b** GGBFS and **c** TLF.

A Rigaku Miniflex diffractometer for X-ray diffraction (XRD) was used to conduct the analyses. The samples' diffraction patterns were gathered and subjectively examined throughout a 2θ range from 5° to 60°. The diffraction patterns of the GGBFS, CCR, and TLF are shown in Fig. 3. GGBFS is amorphous and has a halo between 25° and 35° (2°), whereas CCR is mostly composed of Portlandite and calcite and shows no signs of having any amorphous phases. These alkalis' presence in the CCR will hasten the GGBFS' reactivity. Calcite and quartz make up the LF's powder XRD pattern.

The main oxides present in CCR were lime and silica, identified using the X-ray fluorescence technique (XRF) with a Shimadzu EDX 720 energy dispersive X-ray fluorescence spectrometer, which aligns with the findings of Hanjitsuwan et al.^49^. On the other hand, a chemical analysis of GGBFS, as depicted in Table 1, revealed the presence of lime, alumina, silica, magnesium, and sodium in its composition. The chemical composition results are in agreement with those examined by Chaunsali and Peethamparan^50^. Because CCR contains a significant amount of calcium oxide, it is appealing for use as a promoter similar to OPC^33^. CCR is a possible material for GGBFS activation due to its chemical composition. According to the TLF's chemical compositions, CaO and SiO_2_ made up the majority of their composition. Although TLF contains a lot of CaO (77.82%), it is regarded as an inert substance since CaO doesn't hydrate when it comes in touch with water because it already exists in a non-hydrated condition.

**Table 1:** Comparison of the fillers' chemical composition.

Chemical composition	T LF	GGBFS	CCR
CaO, %	77.82	40.35	81.84
SiO_2_, %	17.21	37.23	14.08
Al_2_O_3_, %	0.0	5.73	0.90
MgO, %	0.89	4.22	0.77
Fe_2_O_3_, %	0.0	0.01	0.00
SO_3_, %	0.01	0.0	0.77
K_2_O, %	0.35	0.0	0.20
Na_2_O, %	2.27	0.0	1.32
TiO_2_, %	0.19	0.63	0.12

**Figure 4:**
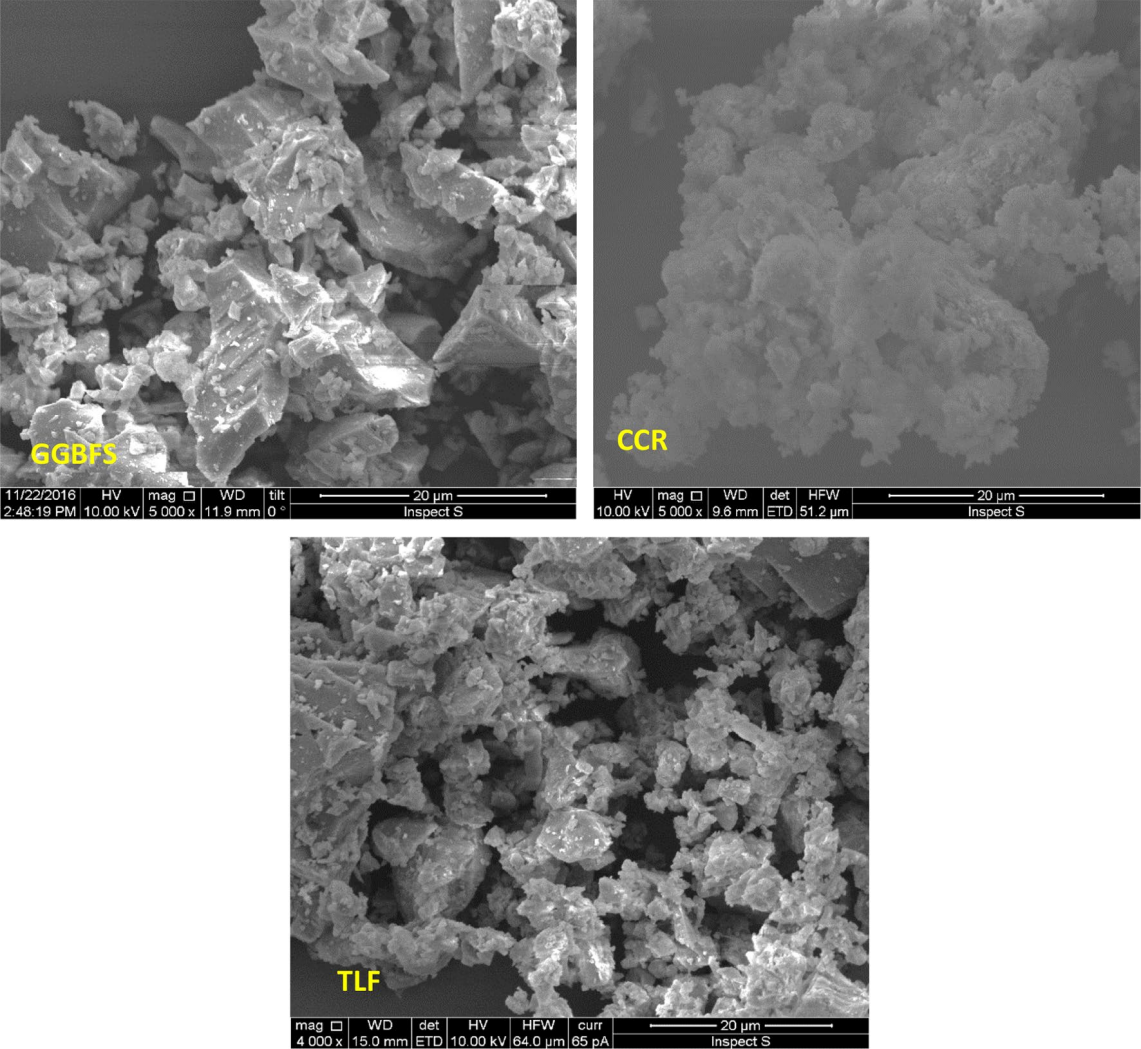
SEM images of the fillers.

Figure 4 displays SEM photos of every filler produced by a Quanta 200 using scanning electron microscopy (SEM). Using an auto fine sputter coater, a tiny layer of gold was applied to the samples to improve conductivity. As can be observed, the CCR particles are agglomerated, whereas the GGBFS particles are primarily made up of irregularly shaped particles. This is similar to the study by Hanjitsuwan et al.^33^. All TLF particles have sharp angles and rough roughness.

The aqueous GGBFS solution had a pH value of 11.6, while the CCR showed a pH of 13.1. This difference in pH levels played a role in the accelerated breakdown of the bitumen emulsion in the CAEMs. On the other hand, the TLF exhibited a pH of 9.39.

### Manufacturing of samples

The mix proportions for the CAEMs are shown in Table 2. It was recommended to use 12.5% bitumen emulsion to produce mixes that would increase the CAEMs' early-age strength. Therefore, the fixed amount of 4% GGBFS + 2% CCR was incorporated in accordance with previous research^51^. The bitumen emulsion used here with 50% base bitumen, and the pre-wetting water contained was 3%. After that, a 3% waste alkaline Ca(OH)_2_ solution replaced the 3% pre-wetting to produce a novel GCAE mix.

**Table 2:** Details on the mix's ratios.

Mix type	Filler types	Bitumen emulsion, %	Pre-wetting water, %
TLF	6% TLF	12.5%	3%
4GGBFS2CCR	4% GGBFS + 2% CCR	12.5%	3%
GCAE	4% GGBFS + 2% CCR	12.5%	(3%) Alkaline waste Ca(OH)_2_ solution
HMA 40/60	6% TLF	5.1% Base binder 40/60	–
HMA 100/150	6% TLF	5.1% Base binder 100/150	–

In accordance with the recommendations of Dulaimi et al.^52^ and following the Marshall Method for Emulsified Asphalt Aggregate Cold Mixture Design (MS-14)^53^, the aggregates, including the coarse and fine components, as well as the fillers, were initially mixed with the required pre-wetting water for 1 min at a slow speed to prepare the cold asphalt emulsion mixtures (CAEMs). The bitumen emulsion was gradually added during the subsequent 30 s of mixing. The mixing process continued for an additional 2 min. Afterwards, the mixtures were quickly poured into steel molds and compacted by applying 50 blows with a Marshall hammer to each surface of the samples.

All samples were then extruded using a hydraulic de-moulding jack after being kept in their molds for 24 h. These mixes were compared with both CAEMs (TLF and 4GGBFS2CCR) and conventional hot mix asphalt mixtures.

### Laboratory testing programme

**Figure 5:**
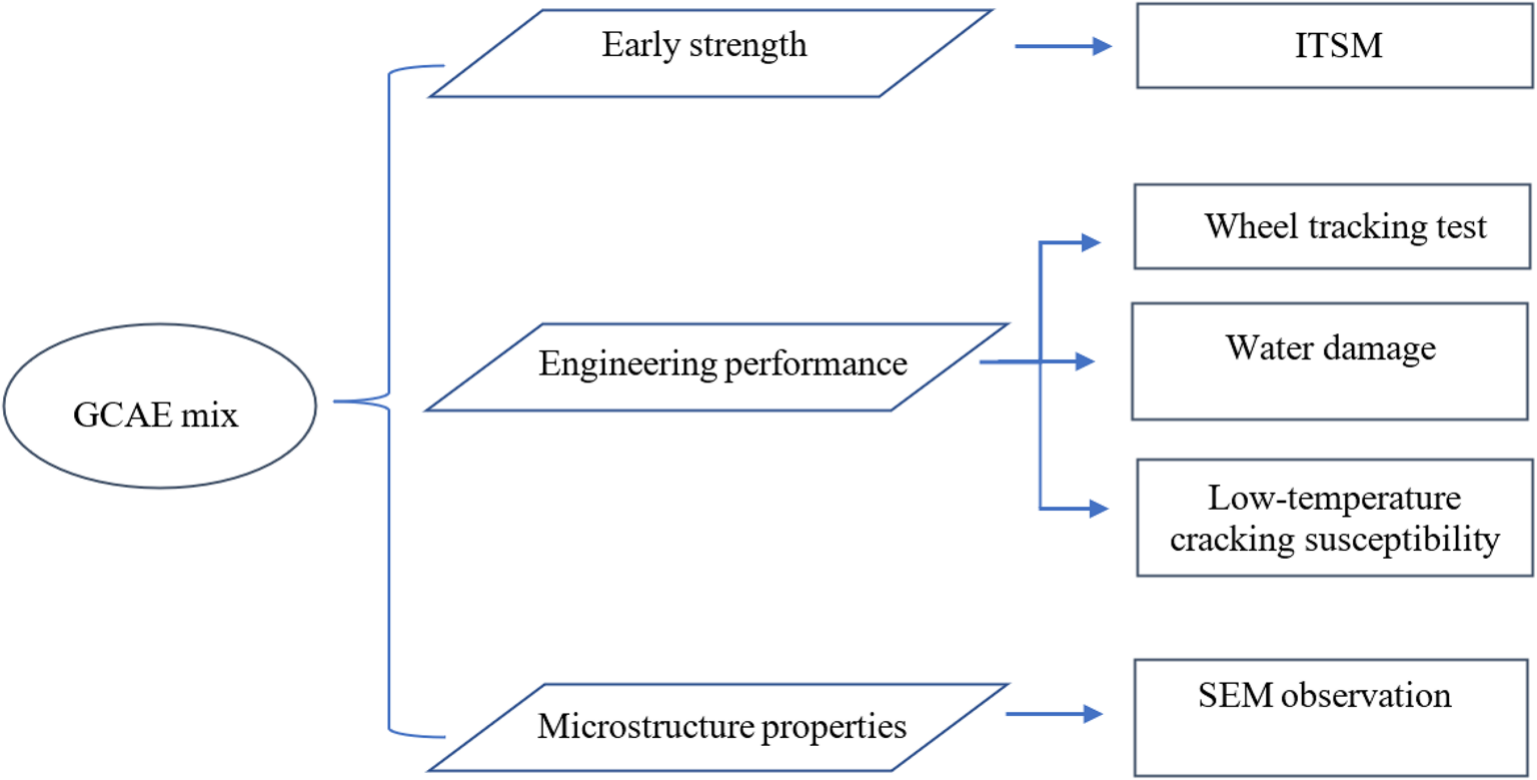
Experimental plans.

This research reports the results of the test of indirect tensile stiffness modulus test (ITSM), wheel truck resistance, water sensitivity and low-temperature cracking susceptibility of a new, novel geopolymer cold asphalt emulsion mixture in addition to the references to traditional CAEMs and HMAs. The procedures for testing emulsified asphalt cold mix (EACM) specimens are shown in Fig. 5.

#### ITSM test

The ITSM was determined at the standard prescribed ambient temperature of (20 ± 1 °C) as per the following BS EN 12697-26^54^, using a Cooper Research Technology HYD 25 testing machine. Three replicates of each sample were created to evaluate the ITSM. The cylindrical sample was generated by applying 50 blows to each side of the sample, extracting it after 24 h, and storing it in the lab at room temperature for testing over the course of 3, 7, 14, 28 and 56 days. When testing for ITSM, the room temperature was chosen as the standard curing temperature for produced mixes. This procedure was followed to simulate the production, placing, and compacting of CAEMs on the site and to avoid premature binder aging^53,54^. The Marshall specimens were 101.6 mm in diameter and 63.5 mm in height. The ITSM of the mixes was evaluated using the average of three replicates.

#### Wheel-tracking test

The assessment of rutting effectiveness was carried out by comparing the performance of GCAE mixes with reference mixes. The evaluation was conducted following European standards outlined in BS EN 12697-22^55^. To measure rut depths, a wheel tracker device of type HYCZ-5 was employed. The rutting slab employed in the testing had dimensions of 400 mm × 305 mm × 50 mm. The average of three replicates was recorded to evaluate the rutting resistance of the mixes. Before conducting the tests, the slab was compacted using a roller compactor, following the standard procedure outlined in BS EN 12697-33^56^. The test temperature was maintained at 60 °C, and a total of 10,000 cycles were conducted to assess the performance of the mixes under high-temperature conditions.

To accurately identify the failure mechanisms of the mixes under controlled conditions, the test was conducted to closely simulate field conditions as much as possible. At a lab temperature of 20 °C, these slabs were held in their molds for 1 day. They were then allowed to cure for an additional day at 40 °C, followed by 21 days at lab temperatures, during which a consistent mass was attained according to the procedure of Thanaya^13^.

#### Low-temperature cracking susceptibility

Utilizing a universal testing machine type (H25KS), a three-point bending test was conducted to evaluate the low-temperature properties of the asphalt mix in accordance with BS EN 12697-44^57^. The test specimens for semi-circular bending (SCB) were first created from slab specimens. After that, the H25KS universal testing apparatus was used to assess how well mixed specimens could withstand cracking at low temperatures. From compacted slabs, several cylindrical specimens with dimensions of 150 mm in diameter and 50 mm in depth were extruded. The loading speed was 50 mm/min, and the temperature was set at 5 °C. The bending strength was determined based on the three-point bending test to assess the mixtures' sensitivity to fracture at low temperatures^58^. To assess the mixes' susceptibility to low-temperature cracking, the average of three replicates was recorded.

#### Water sensitivity test

Moisture has a significant impact on the adhesion between the binder and aggregates in asphalt mixtures, leading to crack formation and surface course weakening^59^. To evaluate the durability of the mixtures, the water sensitivity test was conducted in accordance with the British Standard EN 12697-12^60^. The test aimed to assess the resistance of the mixtures to the effects of water. Additionally, the stiffness modulus ratio (SMR), was determined as a measure of the mixtures' resistance to moisture-induced damage.

The cylindrical samples were manufactured and separated into dry and wet sets. One of the sets was submerged in water heated to a conditioning temperature of 40 °C after being wet. The water level was 25 mm over the top surface of the wet group specimens, which were placed in a water bath at 20 °C for 4 days. They spent 10 min at a pressure of 6.7 kPa inside a vacuum container that was kept at lab temperature. They were held under water for a further 30 min when air pressure was progressively introduced into the vacuum container.

Afterwards, the specimens were immersed in a water bath at a temperature of 40 °C for a duration of 72 h. Following the completion of this water immersion period, the dried samples were placed on a flat surface after undergoing the preparation and compaction phases. Following the conditioning procedures, each group's ITSM and SMR were identified.

$${\text{SMR }} = \, \left( {{\text{ITSM}}_{{{\text{wet}}}} /{\text{ ITSM}}_{{{\text{dry}}}} } \right) \times {1}00$$

where SMR: The stiffness modulus ratio. ITSM_wet_: The indirect tensile stiffness modulus for wet samples. ITSM_dry:_ The indirect tensile stiffness modulus for dry samples. The average of three replicates was recorded to evaluate the water sensitivity of the mixes.

### Microstructures test

SEM images were obtained from small dried samples extracted from fully cured paste specimens after a 28-day curing period. These images were used to analyze the microscopic morphology and pore structure of the hydrated product. A “Quanta 200” scanning electron microscope with a working distance of 13 mm and an accelerating voltage of 10 keV was utilized to capture the images at various magnifications.

## Results and discussion

### ITSM results

It was reported that the practical applications of the CAEM mixes in the need for speedy open traffic can be directly impacted by its early-age strength^58^. In order to acquire a thorough understanding of the CAEM mixture's early-age strength, ITSM was used.

Previous studies have indicated that the optimal combination of GGBFS and CCR in CAEMs is 4% and 2% of the total aggregate mass, respectively^51^. The use of a novel cementitious binary blended filler, consisting of GGBFS and CCR, has shown significant effectiveness in enhancing the properties of CAEMs. CCR, with its high alkalinity (pH = 13.1), serves as the activating medium for GGBFS, which acts as a latent hydraulic material. Subsequently, the pre-wetting water is replaced with a waste alkaline Ca(OH)_2_ solution to produce a geopolymer cold asphalt emulsion mixture (GCAE). It is worth noting that altering the pH of emulsions can result in their destabilization^61^. After a three-day curing period, the GCAE exhibited a maximum stiffness modulus of 2465 MPa, representing a 13-fold improvement compared to the TLF mix, which measured 1678 MPa when incorporating 4% GGBFS + 2% CCR.

**Figure 6:**
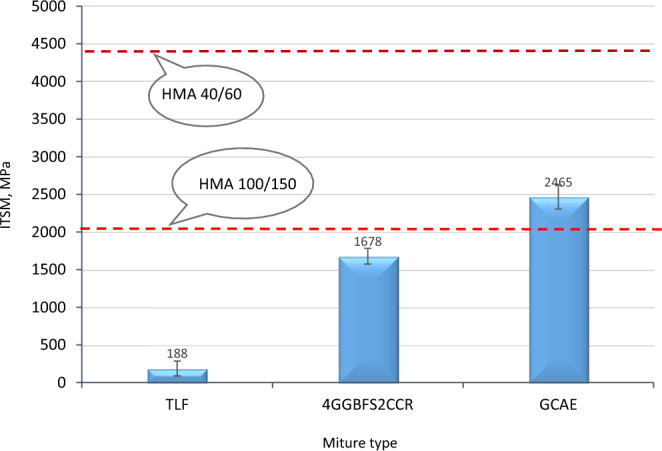
ITSM after 3 curing days.

**Figure 7:**
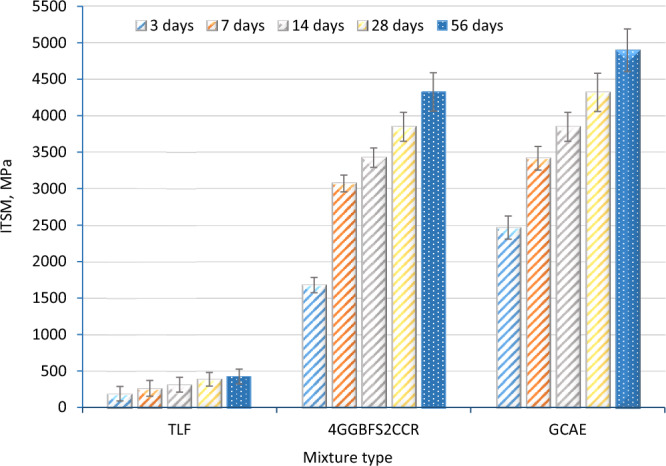
ITSM after 3, 7, 14, 28 and 56 curing days.

Figures 6 and 7 illustrate the progression of the ITSM test results for different mixtures, including TLF, 4GGBFS2CCR, GCAE, and the reference hot mixes, at various curing durations (3, 7, 14, 28, and 56 days). Notably, the ITSM of the GCAE mix exhibited significant improvements across all curing timeframes, surpassing the ITSM of the conventional HMA (100/150 pen) within just 3 days of standard curing.

Remarkably, the ITSM results for these mixes exceeded those of HMA, typically used on heavily trafficked roads, within a mere 3 days. By the 7th day, the GCAE mix demonstrated more than double the performance of HMA 100/150. After 28 days of normal curing, the GCAE mixture exhibited over 110% of the ITSM exhibited by the hard HMA (40/60 pen). This remarkable early-age strength of GCAE can be attributed to the addition of the waste alkaline Ca(OH)_2_ solution. The combination of the waste Ca(OH)_2_ solution and CCR played a crucial role in activating the latent hydraulic material, GGBFS, resulting in this significant enhancement of ITSM. The hydration products formed as a result of this activation process penetrate the binder bond and disperse throughout it, further strengthening the binder^62^. Furthermore, the elevated pH of the hydration medium aided in the dissolution and disintegration of the glassy phase of the pozzolanic material. Moreover, due to their alkaline nature, the waste alkaline Ca(OH)_2_ solution and CCR expedited the breakdown of the cationic bitumen emulsion.

On the other hand, the TLF mix showed inferior performance at all curing ages. The strength development of CAEM primarily relies on two factors: the removal of trapped water and the setting of the bitumen emulsion to its original base bitumen state. Such TLF mix initially showed low strength and gradually acquired some strength with time due to the rising rate of water evaporation. However, compared to the two categories of HMA, its performance is still lacking. It was reported that the early-age strength of the CMA mix can directly affect its practical applications in the requirement of rapid open traffic^58^. It was worth mentioning that the bulk density of the TLF, 4GGBFS2CCR, GCAE, HMA 100/150, and HMA 40/60 were (2.163, 2.175, 2.189, 2.325, and 2.316) g/cm^3^, respectively. However, the proximity of density of above cold mixes has a limited effect on their mechanical and durability properties, while the constitutions of the mixes control these properties.

### Wheel tracking test results

**Figure 8:**
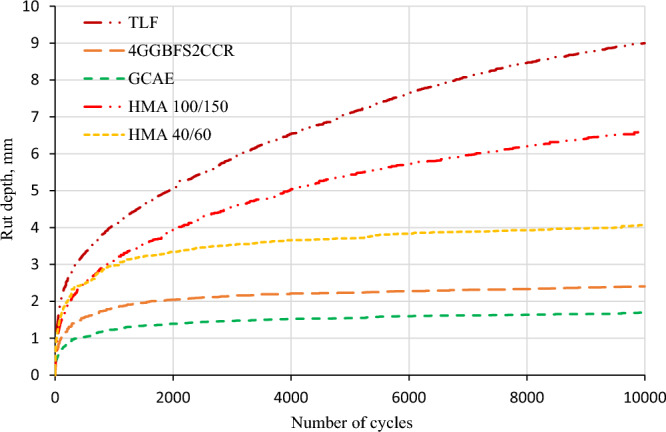
Wheel track results.

The rut depths of each mixture combination were calculated using the wheel tracker test. The rut depth in various mixes reached after 10,000 cycles is shown in Fig. 8 below. The control mix (TLF) had a depth of around 9 mm, which was reduced to 2.4 mm by the addition of 4GGBFS2CCR, and further dropped to 1.7 mm by the addition of the waste alkaline Ca(OH)_2_ solution to the mixtures. Subsequently, by the addition of the waste alkaline Ca(OH)_2_ solution to the filler made from 4GGBFS2CCR, there was a drastic decrease of around 40% in the rut depth in comparison to the 4GGBFS2CCR mix. This shows that the mix had better resistance to rutting susceptibility when the alkaline solution was added.

The reason for this is the improved hydration products. This improves the bonding characteristics of the internal microstructure bonding agent that binds the fine and coarse aggregates. In addition, asphalt emulsion demulsification was sped up by the 4GGBFS2CCR filler. In the meanwhile, the water in the CAEM might promote the hydration process, thus consuming the excess water that normally exists in conventional CAEM, consequently improving the stiffness and rutting resistance of these mixtures^63,64^.

Furthermore, the replacement of pre-wetting water with the waste alkaline Ca(OH)_2_ solution further increased the hydration process. As a result, hydration products as well as asphalt films may be joined to create a network structure that would offer exceptional deformation resistance. Similar behaviour was also reported by Lu et al.^58^.

### Low-temperature cracking susceptibility results

**Figure 9:**
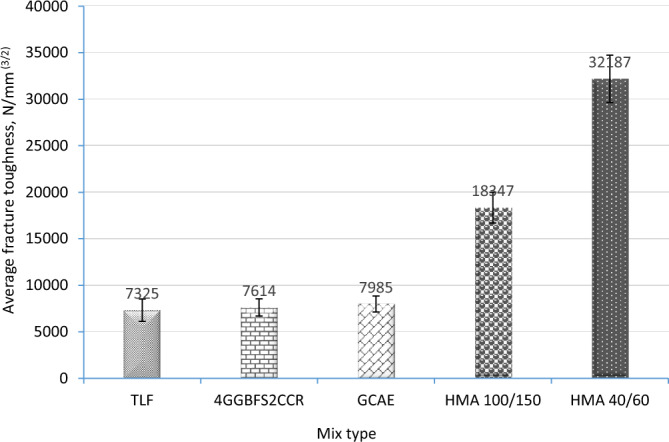
Fracture toughness for the mixes.

The fracture toughness values are presented in Fig. 9. As suggested in this figure, the TLF mix had the lowest bending fracture toughness value. This might be caused by the TLF mix's low ITSM, which has a significant impact on the fracture propagation mechanism. When 4GGBFS2CCR was used, the fracture toughness values of the mixes increased to 7614 N/mm^2/3^, meaning an increment of approximately 4%. This is expected because both GGBFS and CCR increased the stiffness of the mixtures. Additionally, the fracture toughness values could further be increased to about 7985 N/mm^2/3^ for GCAE, which is about 9% higher than that of the TLF mix. This can be attributed to the effect of the increase in ITSM. However, all these mixes have less fracture toughness in comparison to both HMA (100/150 pen) and HMA (40/60 pen).

### Water damage results

**Figure 10:**
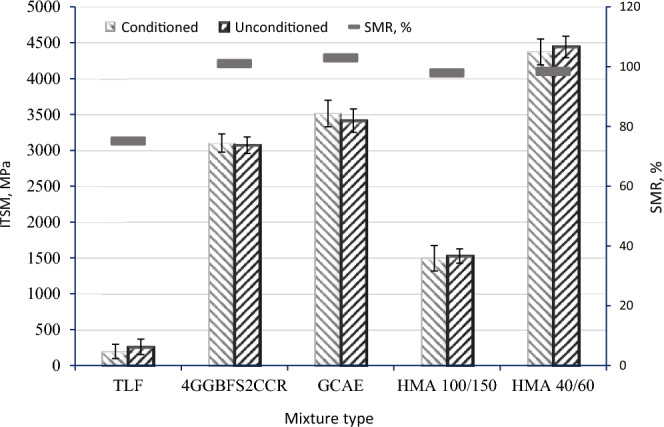
Water damage resistance results.

The ITSM and SMR values of mixes containing the employed ingredients are shown in Fig. 10. It is evident that both the ITSM and SMR values showed an increase when 4GGBFS2CCR (4% GGBFS + 2% CCR) were utilized. Furthermore, the incorporation of the waste alkaline Ca(OH)_2_ solution resulted in the highest values for both ITSM and SMR.

Its SMR value for the TLF mix was only 75%. The cementitious filler 4GGBFS2CCR substantially raised the SMR value to 101%. Similar to this, the waste alkaline Ca(OH)_2_ solution that was integrated showed a higher SMR value of 103%. Other reports have supported similar outcomes^58^. This was mostly due to the use of GGBFS and CCR that were activated by the waste alkaline Ca(OH)_2_ solution, which produced many hydration products that increased the strength and stiffness of the GCAE. These findings align with the research conducted by Tian et al.^65^, which demonstrated improved water susceptibility when incorporating a high content of cement in CAEMs. The advantage of introducing GGBFS, CCR, and the waste alkaline Ca(OH)_2_ solution increased the formation of hydration products and enhanced water susceptibility.

### SEM observation

**Figure 11:**
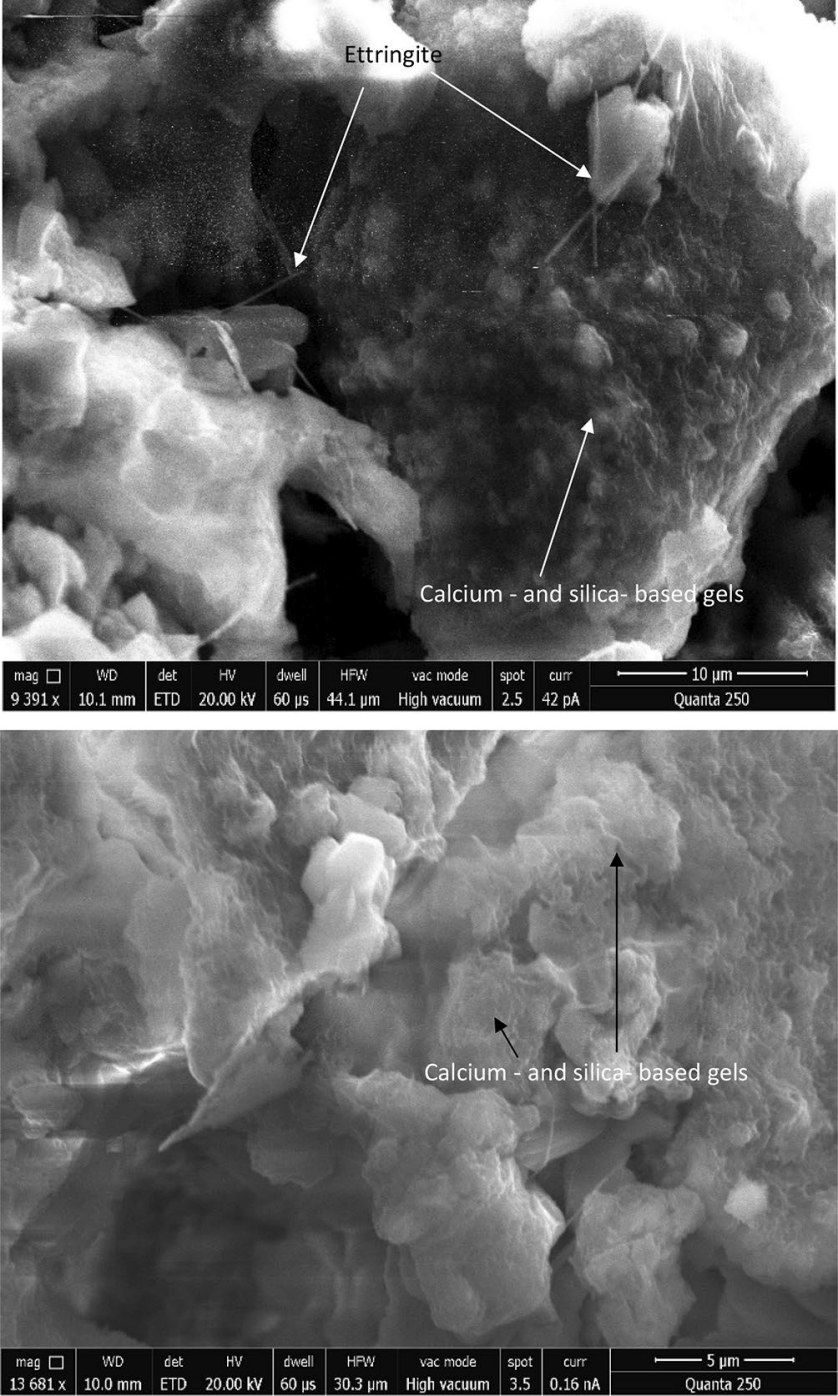
The morphology details of the microstructures of GCAE at 28 days.

SEM analysis was conducted on the samples to confirm the development of cementitious products in the 4GGBFS-2CCR geopolymer over time. The formation of a dense cementation matrix can be attributed to the chemical reaction between SiO_2_, Al_2_O_3_, and MgO (present in GGBFS) and CaO and SiO_2_ (present in CCR), which effectively binds the particles together. The SEM images reveal the presence of a more continuous matrix with a denser structure. Notably, Fig. 11a and b indicate the absence of a residual shell formed by large-sized GGBFS and CCR particles, indicating their erosion by the alkaline solution.

The SEM analysis reveals that the pozzolanic reactions, containing calcium- and silica- based gels and needles fill the pore structure and contribute to the increased density of the mixture. The microstructure appears dense, with the presence of various cement hydrates and no visible voids. As a result, the paste sample exhibits a dense microstructure and higher strength. Furthermore, upon closer inspection, the surface of the hardened paste specimen appears smooth without any uneven protrusions. The formation of calcium- and silica- based gels and needles contributes to the densification of the matrix. According to Shi and Day^66^, the initial pH of the activating medium plays a crucial role in the alkali-activated slag process, influencing the early reaction products’ formation and the dissolution of slag. Consequently, the performance properties of GCAE are enhanced.

The dense structure of the calcium- and silica- based gels and needles and asphalt films contributes to the formation of a network structure, leading to improved strength in GCAE. The predominant presence of the calcium- and silica- based gels phase contributes to the regular and compact microstructure. Additionally, under further magnification, needle-like crystals can be observed filling the voids left by water evaporation. The exceptional early-age strength performance of GCAE mixes can be attributed to the evident densification of the microstructure. These findings align with previous studies conducted by Lu et al.^58^ and Dulaimi et al.^46^.

## Conclusions

Based on the research findings regarding the mechanical properties, work performance, and hydration characteristics of the geopolymer cold asphalt emulsion mixture (GCAE) incorporating waste alkaline Ca(OH)_2_ solution, ground granulated blast-furnace slag (GGBFS), and calcium carbide residue (CCR), the following conclusions can be drawn:

1. Because of the considerable curing period required for CAEM to acquire full strength after paving, the use of such mixes using TLF as structural layers is not feasible.
2. The waste alkaline Ca(OH)_2_ solution is favorable to enhance the mechanical properties of GCAE. The substitution of such material causes the increase of hydration product content. Furthermore, such a solution can improve the performance properties of GCAE.
3. The GCAE mixture exhibits enhanced early-age strength, as indicated by an increased stiffness modulus. It outperforms traditional hot mix asphalt (HMA) within a short curing period, demonstrating its potential for high-performance applications. The ITSM increased due to the addition of waste alkaline Ca(OH)_2_ solution along with both GGBFS and CCR fillers. Notably, the ITSM of the GCAE in this study increased by approximately 13 times compared to the traditional LF mix after 3 days of curing. The addition of waste alkaline Ca(OH)_2_ solution significantly contributed to outstanding early-age strength.
4. The rut depth was significantly lower compared to the reference TLF mix due to the utilization of waste alkaline Ca(OH)_2_ solution, GGBFS, and CCR fillers, resulting in rut depths 5.5 times shallower than the traditional LF mix. Additionally, there was a substantial improvement in rutting resistance, with increases of 2.5 and 4 times compared to traditional HMA 100/150 and HMA 40/60, respectively. This suggests that these waste materials substantially enhance material stiffness while also reducing rut depths at high temperatures. Therefore, it is well-suited for use in road pavements exposed to challenging service conditions, including temperature variations.
5. Early-age GCAE strength was high and rapidly developed in the waste alkaline Ca(OH)_2_ solution. After 3 days of curing, the ITSM from such mix reached more than 2465 MPa and was roughly 50% of what it was at 28 days, demonstrating that this design approach may be used to produce GCAE with exceptional early-age strength.
6. The increase in the stiffness modulus ratio (SMR) was also observed when the waste alkaline Ca(OH)2 solution replaced pre-wetting water in the mixtures, showing higher resistance to moisture. Notably, the SMR value exhibited an approximately 40% increase compared to the traditional LF mix. Furthermore, the SMR increase exceeded 5% when compared to hot mix asphalt (HMA). This suggests that the waste alkaline Ca(OH)_2_ solution, in combination with GGBFS and CCR fillers, added to the mixtures, provided both high stiffness and strong bonding.
7. The asphalt emulsion was demulsified with the use of the waste alkaline Ca(OH)_2_ solution, GGBFS, and CCR fillers, resulting in a much higher amount of hydration products and a regular, compact microstructure. The excellent stiffness has, nevertheless, resulted in a minor improvement in the cracking susceptibility.
8. The utilization of GGBFS and CCR as fillers in the GCAE mixture contributes to the formation of a dense cementation matrix. This matrix, formed by the reaction between various compounds in GGBFS and CCR, improves the binding between particles and results in a stronger and more durable pavement structure.

These findings suggest that the incorporation of waste alkaline Ca(OH)_2_ solution, GGBFS, and CCR in the geopolymer cold asphalt emulsion mixture offers a promising solution for achieving high-performance and sustainable asphalt pavements. The following steps will be implemented at the construction site, and we recommend the use of GCAE mix to enhance the initial road performance. Further research and development in this area can lead to the utilization of industrial by-products and the production of more durable and environmentally friendly road materials.

## Limitations and novelty of research

While this study has provided valuable insights and promising results, it is crucial to recognize its limitations. It’s worth noting that certain tests within this research had relatively short time frames. A more extended evaluation of performance and durability in the long term may uncover further insights and potential challenges not addressed in this study. Furthermore, the experiments were carried out within controlled laboratory settings, which may not fully account for real-world conditions, including traffic loads and environmental factors, which can introduce additional complexities.

In spite of its limitations, this research makes several noteworthy contributions. The incorporation of waste alkaline Ca(OH)2 solution, combined with GGBFS and CCR fillers in asphalt mixtures, introduces an innovative approach to enhancing sustainability in road construction. The substantial enhancements in stiffness, rutting resistance, and bonding observed in this study underscore the potential for these waste materials to elevate asphalt mixture performance. Moreover, the research underscores the efficacy of the waste alkaline Ca(OH)_2_ solution in enhancing moisture resistance, a critical factor in road pavement durability.

## Abbreviations

CAEM: Cold asphalt emulsion mixture
CBEM: Cold bitumen emulsion mixture
CCR: Calcium carbide residue
CMA: Cold mix asphalt
EACM: Emulsified asphalt cold mix
GCAE: Geopolymer geopolymer-based cold asphalt emulsion mixture
GGBFS: Ground granulated blast-furnace slag
HMA: Hot mix asphalt
ITSM: Indirect tensile stiffness modulus test
LF: Limestone filler
OPC: Ordinary Portland cement
SCB: Semi-circular bending
SCM: Secondary cementitious material
SEM: Scanning electron microscopy
SMR: Stiffness modulus ratio
TLF: Traditional limestone filler
WMA: Warm mix asphalt
XRD: X-ray diffraction
XRF: X-ray fluorescence technique
